# Supplementary material for: Identification of Genomic Safe Harbors in the Anhydrobiotic Cell Line, Pv11
Source: Genes (Basel). 2022 Feb 24;13(3):406. doi: 10.3390/genes13030406 (PMC8949610; doi:10.3390/genes13030406)
Supplement: Supplementary file 1 [file genes-13-00406-s001.zip › genes-1607004-supplementary.pdf]

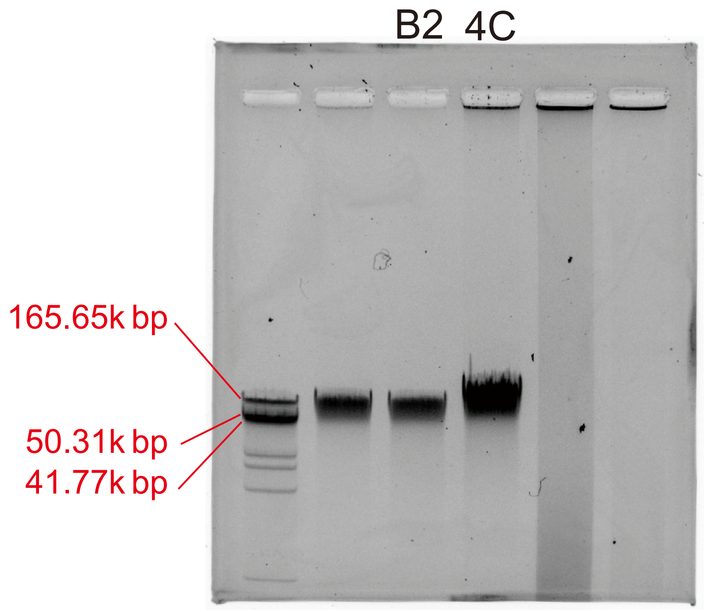

Figure S1. Agarose gel electrophoresis image of extracted genomic DNAs. Fragmented short DNAs were eliminated from the extracted DNAs, and the samples were subjected to library preparation and subsequent MinION sequencing.

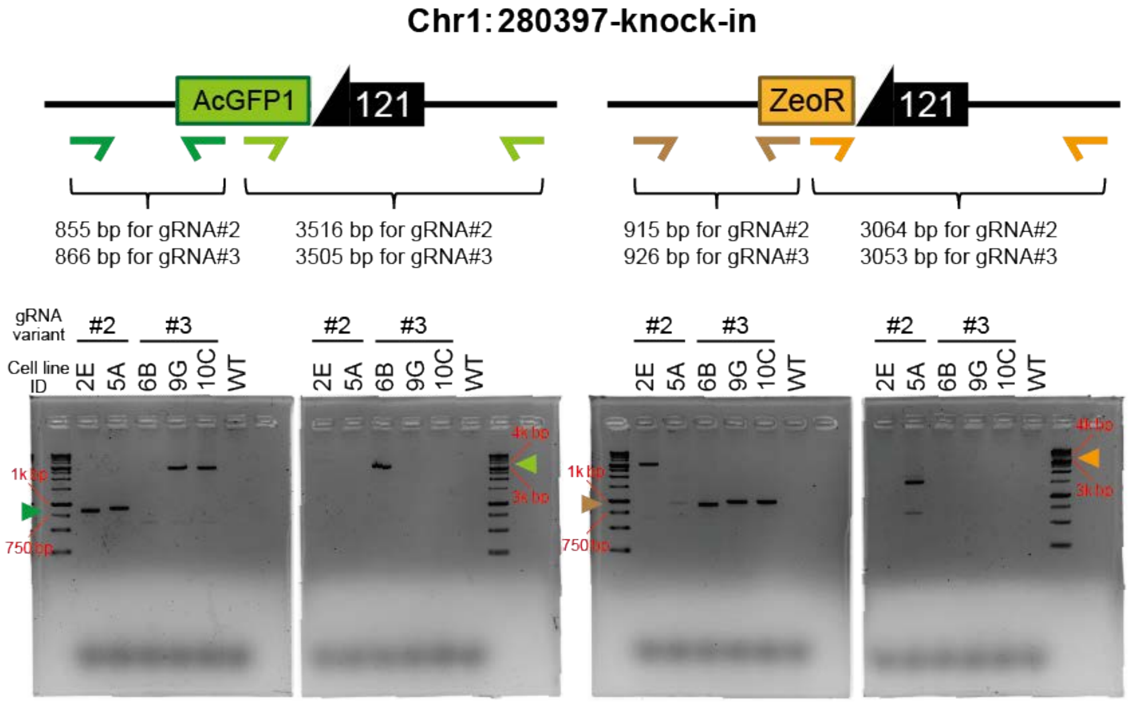

Figure S2. Agarose gel electrophoresis image of genomic PCR in knock-in cell lines at the Chr1:280397 site. Two types of gRNA-expression vectors were used for establishing the knock-in cell line at the Chr1:280397 site. Arrows with the same color indicate a primer set for the genomic PCR, and each expected band size was described below and shown on the gel with an arrow head.

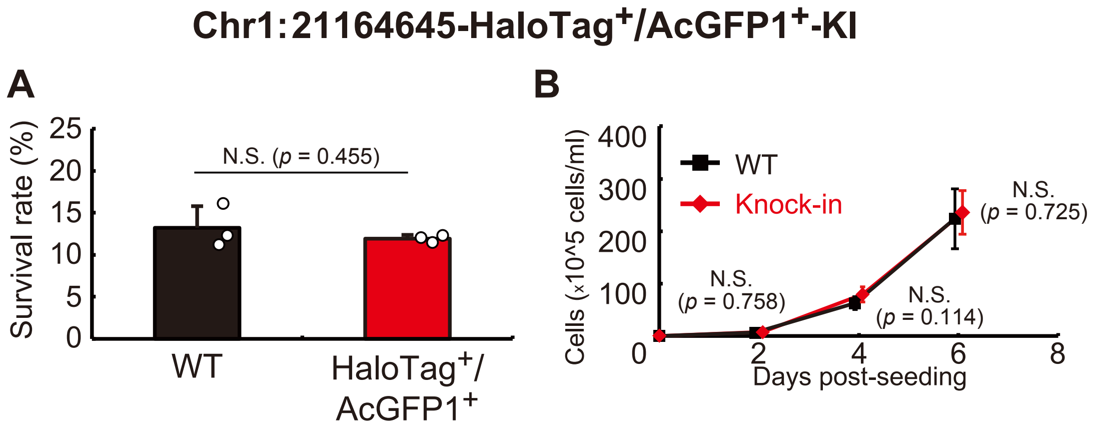

Figure S3. The anhydrobiotic ability and the proliferation rate in the knock-in cells of a GOI-expression unit. In addition to the knock-in efficiency shown in Figure 5, further analysis of the phenotypes was carried out. Values are expressed as mean ± SD; n = 3 and 4 in A and B, respectively, in each group.

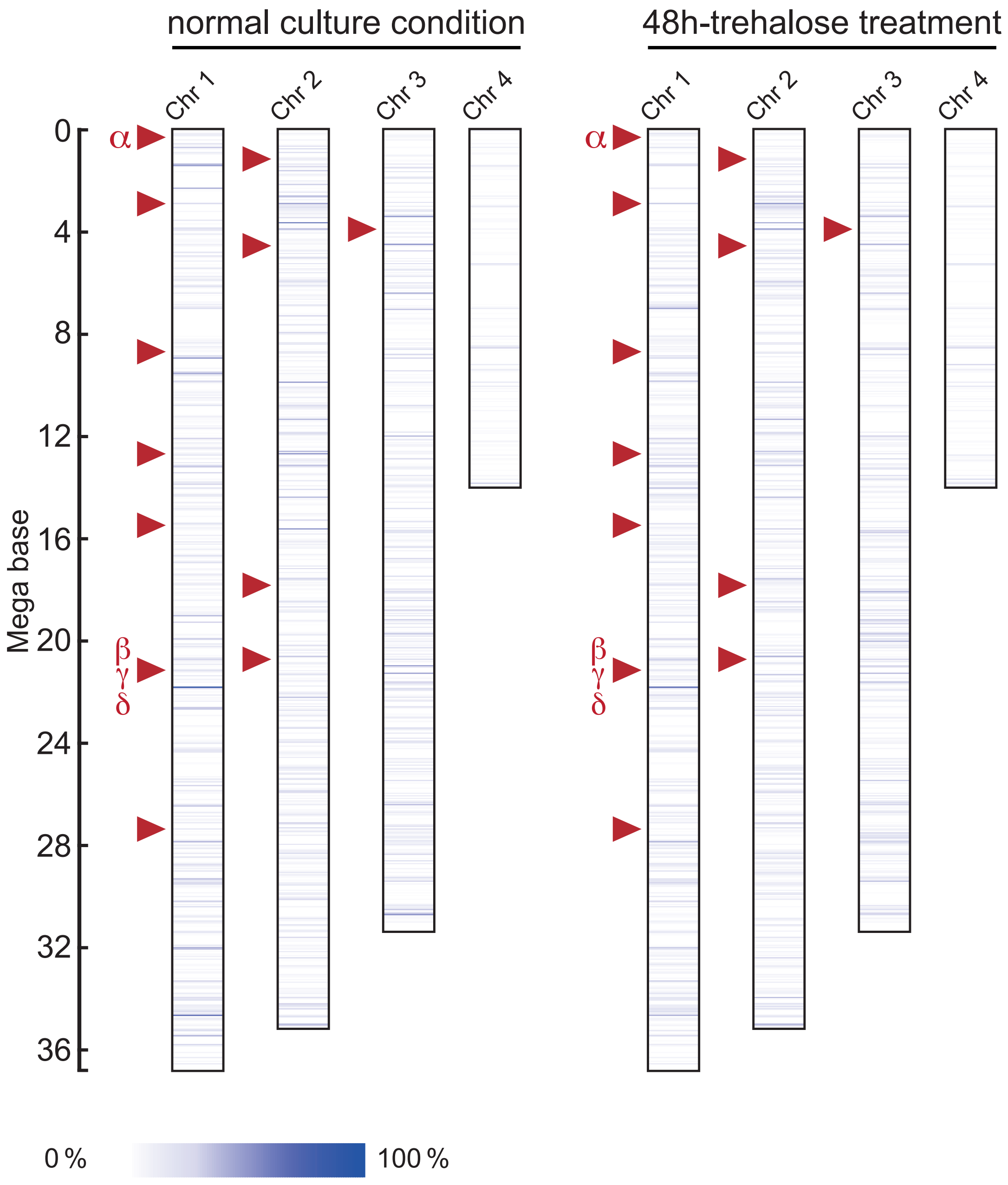

Figure S4. Visualization of ATAC-seq peaks. Arrow heads and alphabets indicate the integration sites of the transfected plasmid and the AcGFP1-expression unit, respectively, in the B2 or 4C lines as shown in Figure 2. ATAC-seq was performed, following the previously reported protocol (Buenrostro *et al.*, 2015). Briefly, 75,000 cells were centrifuged at 500 g for 5 minutes at 4°C, and washed once with cold PBS and 600 mM trehalose solution for normal condition and trehalose-treated samples, respectively. The cells were resuspended in lysis buffer for 10 minutes. Nextera DNA Sample Prep Kit (Illumina) was used for the following tagmentation reaction, and the products were purified with MinElute PCR Purification Kit

(Qiagen). For preliminary amplification of the libraries, five cycles of PCR were performed with primers from the original protocol (Buenrostro *et al.*, 2015), and further seven cycles of PCR were also done as the final amplification. The ATAC-seq libraries were purified with Agencourt AMPure XP beads (Beckman Coulter), and subsequently sequenced by Illumina paired-end reads 100 bp in a HiSeq 2500.

Buenrostro JD, Wu B, Chang HY, Greenleaf WJ. ATAC-seq: A Method for Assaying Chromatin Accessibility Genome-Wide. Curr Protoc Mol Biol. 2015 Jan 5;109:21.29.1-21.29.9. doi: 10.1002/0471142727.mb2129s109. PMID: 25559105; PMCID: PMC4374986.

Table S1. Oligo DNA sequences for construction of gRNA-expression vectors.

Vector name	Oligo name	Sequence (5' to 3')
pPvU6b-DmtRNA-280397#2	chr1_280397-gRNA#2-sense	tgcaTATTCAAGGAGGTACAAAAA
	chr1_280397-gRNA#2-antisense	aaacTTTTTGTACCTCCTTGAATA
pPvU6b-DmtRNA-280397#3	chr1_280397-gRNA#3-sense	tgcaAATCTTTAGTTTATTCAAGG
	chr1_280397-gRNA#3-antisense	aaacCCTTGAATAAACTAAAGATT
pPvU6b-DmtRNA-21143572#5	chr1_21143572-gRNA#5-sense	tgcaCTGTCAGTAACTTCAGTGAC
	chr1_21143572-gRNA#5-antisense	aaacGTCACTGAAGTTACTGACAG
pPvU6b-DmtRNA-21155382#2	chr1_21155382-gRNA#2-sense	tgcaGTTATACACAAGACAAATTG
	chr1_21155382-gRNA#2-antisense	aaacCAATTTGTCTTGTGTATAAC
pPvU6b-DmtRNA-21164645#9	chr1_21164645-gRNA#9-sense	tgcaAAAAAAATAATCGGCCTGTA
	chr1_21164645-gRNA#9-antisense	aaacTACAGGCCGATTATTTTTTT

Table S2. Primers for genomic DNA sequencing.

Amplified region	Oligo name	Sequence (5' to 3')
chr1_280397	TA_chr1_280397_F1	GAAACGCTCGCTATGAGACC
chr1_280397	TA_chr1_280397_R1	TTCCGAAGACACATGTGGAA
chr1_21143572	YM_chr3_21143572-F3	CAGGCAGAGGCGAGAATGAA
chr1_21143572	YM_chr3_21143572-R3	ACGCTAAAGATACCCGGTGTG
chr1_21155382	TA_chr1_21153515_F1	GGAGTTGGAACCGAAGATGA
chr1_21155382	TA_chr1_21153515_R1	CCCTTTTCTGTCTTGCCTTG
chr1_21164645	YM_chr3_21164645-F3	ACAAAACATCTGAGTGCATGGA
chr1_21164645	YM_chr3_21164645-R3	AATTGCTCTTTCAACCACTCCT

Table S3. Primers for construction of the donor vectors used in Figure 3.

Vector name	Oligo name	Sequence (5' to 3')
pCR4-280397#2uH-121-AcGFP1	YM_280397#2-poly(A)-PITCh_F	TATTCAAGGAGGTACAAAAAAGGCTCATTCACTCTCAGAACCATTTTTATGATTTTACCTTTTCACGCGCTTGAAAGGAGTG
&		
pCR4-280397#2uH-121-ZeoR	YM_280397#2uH-121-PITCh_R	TATTCAAGGAGGTACAAAAAAGGCAGAAAAAAATTAATCTTTAGTTTATTCAAGGAGGTACAACTTCAATTATGATACATGAATAAACA
pCR4-280397#3uH-121-AcGFP1	YM_280397#3-poly(A)-PITCh_F	AATCTTTAGTTTATTCAAGGAGGTCAGAACCATTTTTATGATTTTACCTTTTTTGTACCTCCTCACGCGCTTGAAAGGAGTG
&		
pCR4-280397#3uH-121-ZeoR	YM_280397#3uH-121-PITCh_R	AATCTTTAGTTTATTCAAGGAGGGAAAAAAGTAGCAGAAAAAAATTAATCTTTAGTTTATTCACTTCAATTATGATACATGAATAAACA
pCR4-21143572#5uH-121-AcGFP1	YM_21143572#5-poly(A)-PITCh_F	CTGTCAGTAACTTCAGTGACTGGGGATTAATGCGCAAAAAAGTTCACTGTCAGTAACTTCAGTCACGCGCTTGAAAGGAGTG
&		
pCR4-21143572#5uH-121-ZeoR	YM_21143572#5uH-121-PITCh_R	CTGTCAGTAACTTCAGTGACTGGAAAAATCCACAACGTGAAATTCTTTTTTCTAACGCCAGTCCTTCAATTATGATACATGAATAAACA
pCR4-21155382#2uH-121-AcGFP1	YM_21155382#2-poly(A)-PITCh_F	GTTATACACAAGACAAATTGCGGTTCGTTTGTTGTTGCCAAAAAAAGTTATACACAAGACAAACACGCGCTTGAAAGGAGTG
&		
pCR4-21155382#2uH-121-ZeoR	YM_21155382#2uH-121-PITCh_R	GTTATACACAAGACAAATTGCGGAATTTACAGAAGATTTTTTTTGGTTTCTTATCTTCCGCAACTTCAATTATGATACATGAATAAACA
pCR4-21164645#9uH-121-AcGFP1	YM_21164645#9-poly(A)-PITCh_F	AAAAAAATAATCGGCCTGTAAGGTTTGCCGATATTTATGAGCCTTGAAAAAAATAATCGGCCTCACGCGCTTGAAAGGAGTG
&		
pCR4-21164645#9uH-121-ZeoR	YM_21164645#9uH-121-PITCh_R	AAAAAAATAATCGGCCTGTAAGGTGCGGCAGATGATCGATTCGTTGGAAATGTGAAGCCTTACCTTCAATTATGATACATGAATAAACA

Table S4. Primers for construction of the donor vectors used in Figure 5.

Vector name	Oligo name	Sequence (5' to 3')
pCR4-21164645#9_1kbpHA-SpeI_NotI	YM_pCR4-21164645#9-1kbLHA_fwd	AATTAACCCTCACTAAAGGGAAAAAAATAATCGGCCTGTAAGGAAAAATGCTAATTTAAATTTGTTTCAAAATTG
	21164645#9RHA-Spe_Not-LHA_rev	GAAGCCTTACGCGGCCGCcttcACTAGTAGGCCGATTATTTTTTTCAAG
	21164645#9RHA-Spe_Not-LHA_fwd	TAATCGGCCTACTAGTgaagGCGGCCGCGTAAGGCTTCACATTTCC
	YM_pCR4-21164645#9-1kbRHA_r2	TATAGGGCGAATTGAATTTAAAAAAAAATAATCGGCCTGTAAGGAAAATGCGATTCCTGTCAC
pCR4-21164645#9_750bpHA-SpeI_NotI	YM_pCR4-21164645#9-750bLHA_fwd	AATTAACCCTCACTAAAGGGAAAAAAATAATCGGCCTGTAAGGATTTTTCTTCCCAAAAGCTTATAG
	YM_pCR4-21164645#9-750bRHA_r2	TATAGGGCGAATTGAATTTAAAAAAAAATAATCGGCCTGTAAGGAATTGAAAGTGGGATTTATACC
pCR4-21164645#9_500bpHA-SpeI_NotI	YM_pCR4-21164645#9-500bLHA_fwd	AATTAACCCTCACTAAAGGGAAAAAAATAATCGGCCTGTAAGGTGTAGAATAGAAATAACGTAAATTAC
	YM_pCR4-21164645#9-500bRHA_r2	TATAGGGCGAATTGAATTTAAAAAAAAATAATCGGCCTGTAAGGTTAATGAAGAAAATCAACAAGAC
pCR4-21164645#9_250bpHA-SpeI_NotI	YM_pCR4-21164645#9-250bLHA_fwd	AATTAACCCTCACTAAAGGGAAAAAAATAATCGGCCTGTAAGGTTCTGCAATTTATGGAGC
	YM_pCR4-21164645#9-250bRHA_rev	TATAGGGCGAATTGAATTTAAAAAAAAATAATCGGCCTGTAAGGATTTTTATTTTTCGCTTTTGATTTC
pCR4-21164645#9_125bpHA-SpeI_NotI	YM_pCR4-21164645#9-125bLHA_fwd	AATTAACCCTCACTAAAGGGAAAAAAATAATCGGCCTGTAAGGAAGTCTCAGACATAAAAAGG
	YM_pCR4-21164645#9-125bRHA_rev	TATAGGGCGAATTGAATTTAAAAAAAAATAATCGGCCTGTAAGGTTTACCCATTTATAAATAATTTATAAACG
pCR4-21164645#9_40bpHA-SpeI_NotI	YM_pCR4-21164645#9-40bLHA_fwd	AATTAACCCTCACTAAAGGGAAAAAAATAATCGGCCTGTAAGGTTTGCCGATATTTATGAGCCTTG
	YM_pCR4-21164645#9-40bRHA_rev	TATAGGGCGAATTGAATTTAAAAAAAAATAATCGGCCTGTAAGGTGCGGCAGATGATCGATTC
pCR4-21164645#9_0bpHA-SpeI_AcGFP1_NotI	YM_pCR4-21164645#9-Spe-GFP_fwd	AATTAACCCTCACTAAAGGGAAAAAAATAATCGGCCTGTAAGGACTAGTATGGTGAGCAAGGGCGCC
	YM_pCR4-21164645#9-Not-GFP_rev	TATAGGGCGAATTGAATTTAAAAAAAAATAATCGGCCTGTAAGGGCGGCCGCTTACTTGTACAGCTCATCCATGCCG
pCRII-SpeI-HaloTag-121-121-AcGFP1-NotI	YM_pCRII-SpeI-rOpIE2_fwd	GAATACTCAAGCTATGCATCactagtCACGCGCTTGAAAGGAGTG
	HF-chr3_21154645#9-tagRFP-rev	ATTTCACAAATAAAGCATTTTTTTCACTGCATTCTAGTTGTG
	HF-chr3_21154645#9-AcGFP1-fwd	AAATGCTTTATTTGTGAAATTTGTGATGCTATTGCTTTATTTGTAAC
	YM_pCRII-NotI-OpIE2_rev	CTATAGGGCGAATTGGGCCCgcggccgcCACGCGCTTGAAAGGAGTG

Table S5. Number of reads in the course of the analysis.

Number of reads	B2	4C
Total sequence read after NanoFilt	183,676	107,159
Number of reads containing vector fragments	208	82
Number of reads containing Gapdh promoter + AcGFP1 full length (the expression unit)	100	38
Number of successfully mapped reads containing Gapdh promoter + AcGFP1 full length (the expression unit)	19	33

Table S6. Integration sites of the fragmented plasmid sequences.

Cell line	Integration site	Genomic feature
B2	Chr 1:280397	intergenic region between g9127 and g9128
	Chr 1:2872737	intergenic region between g9428 and g9429
	Chr 1:12663137	intergenic region between g10915 and g10916
	Chr 1:15462478	intron of g11291
	Chr 1:19359941	intergenic region between g11929 and g11930
	Chr 1:21143572	intron of g12121
	Chr 1:21155382	intergenic region between g12122 and g12123
	Chr 1:21164645	intergenic region between g12124 and g12125
	Chr 1:27392178	intron of g12995
	Chr 1:36576632	intergenic region between g14311 and g14312
	Chr 2:1119392	intron of g4308
	Chr 2:1121015	intergenic region between g4309 and g4310
	Chr 2:4538747	intergenic region between g4738 and g4739
	Chr 2:17806787	intergenic region between g6612 and g6613
	Chr 2:20731784	intron of g7027
	Chr 3:3868253	intergenic region between g518 and g519
	Chr 3:29481216	intergenic region between g4004 and g4005
4C	Chr 1:8668683	intergenic region between g10292 and g10293
	Chr 1:12663144	intergenic region between g10915 and g10916
	Chr 1:21143572	intron of g12121
	Chr 1:21155382	intergenic region between g12122 and g12123
	Chr 1:21158910	intergenic region between g12124 and g12125
	Chr 1:21164645	intergenic region between g12124 and g12125
	Chr 2:1119392	intron of g4308
	Chr 2:1121015	intergenic region between g4309 and g4310
	Chr 2:4538747	intergenic region between g4738 and g4739
	Chr 2:17807291	intergenic region between g6612 and g6613
	Chr 3:3868253	intergenic region between g518 and g519

Table S7. TPM values of genes around GSHs and the annotations.

Gene ID	WT						Annotation
	T0 (TPM)			T48 (TPM)			
g9127	39.96274741	44.38486143	45.11384393	108.9899909	100.5851762	101.7583433	Probable phosphomannomutase
g9128	8.270741576	9.962906917	9.93412406	31.24010876	26.06035589	27.56306745	Threonylcarbamoyladenosine tRNA methylthiotransferase
g12121	35.03837289	39.67757944	33.77958261	32.62864612	33.09891273	30.42798138	Putative Sterol regulatory element-binding protein cleavage-activating protein
g12122	240.435078	242.7605787	240.5581022	168.1111558	163.1966174	160.2230713	ATP synthase (F1-ATPase), gamma subunit
g12123	193.7573603	199.2849712	194.4340001	101.4893752	105.1604862	94.84559703	Peroxisomal multifunctional enzyme type 2
g12124	16.6154543	15.12568854	19.83390413	13.41047625	10.48026268	12.26760951	Putative Phospholipid phosphatase homolog 1.2-like protein
g12125	22.10603794	24.61180715	24.03762173	5.909033683	5.482021641	5.188948049	Ca(2+)/calmodulin-responsive adenylate cyclase
g7775 (Pv.00443)	105223.4789	100831.9426	102457.0509	53162.676	54203.24735	54506.29692	
